# Dermatology

**Published:** 1897-01-30

**Authors:** 


### DERMATOLOGY.

**Eczema.**—Shoemaker[1] insists upon the importance of feeding eczematous infants with sterilised milk, since gastro-intestinal catarrh, dependent upon improper nourishment, is often the cause of the disease. At the same time he gives a powder of calomel gr. 1, and magnesia-carbonate gr. 10, every other night. Locally, after the crusts have been removed by poultices, the following ointment should be applied: ℞. Acid carbolici ♏ 5, pulv. camphor gr. 5, sulphuris subl. gr. 10, ung. zinci oxidi, ung. aq. rosæ a. a. ℥ ijs. M.

Siebersohn[2] recommends the treatment of eczema by steam, which not only removes all the crusts, but also assists reabsorption of the infiltrations, and stops purulent secretion from the ulcerated surfaces. A special apparatus, heated by means of a spirit lamp, is employed, and the temperature of the steam is sufficiently cooled before it reaches the skin, so as to avoid scalding. The duration of each application varies from a quarter to half an hour. He has applied this mode of treatment to eczema which was rebellious to all other methods, and it has given excellent results in all cases.

Brocq[3] describes a circumscribed eczema-form eruption, due to malarial poisoning, in a woman aged forty who had previously suffered from eczema on the hand. She developed a small plaque of irregular contour upon the end of the nose, the base of which was red, slightly swollen and congested, and on the surface there were seven or eight greyish-yellow papulo-vesicles. It had lasted about a month without much extension, but every morning, commencing at four or five o'clock, the lesion became very much inflamed, with considerable oozing from the vesicles; this reached its maximum between seven and nine and then gradually diminished until about two o'clock, when the oozing ceased and the inflammation disappeared, and by six o'clock the lesion was scarcely visible. Although the patient had never had true intermittent fever, she had had various manifestations of paludism, which had yielded only to quinine in large doses, and, moreover, had lived seven years in a place where malaria was prevalent. At the end of four days' treatment by quinine the lesion was no longer elevated, and the morning congestive attacks had ceased. On discontinuing this treatment the eruption returned, but again yielded to quinine.

Baumel[4] describes two cases of Dermatitis Exfoliativa Neonatorum, and discusses the etiology of the disease. A certain amount of desquamation is natural after birth, but this may be aggravated by disturbance of the gastro-intestinal function caused by injudicious use of medicines, or by absorption of pathogenic organisms after the separation of umbilical cord. Defective nutrition also plays a part in the production of the cutaneous phenomena.

**Lupus.**—Gilchrist and Stokes[5] describe a case clinically resembling tuberculosis verrucosa of the skin. The lesion was situated behind the left ear, where it had existed about twelve months. The authors exclude tuberculosis as no tubercle bacilli were discovered, and on account of the position, since the tuberculous wart is usually only found on the hands, arms, and feet. There were, however, miliary abcesses in the epidermis, and in these, as well as among the granulation cells in the corium, they found an organism which they classified as an oidium. The authors conclude that the disease was due to the presence and growth of this parasite in the skin.

Tessile[6] reports two cases of lupus successfully treated with Maragliano's serum. This was injected in alternate daily doses of 1 and 5 cc., 105 cc. being injected in one case, in which a complete cure was obtained. In another case of old-standing lupus of the hand only 25 cc. were injected, but the part was covered with gauze impregnated with the serum, and was also cured.

Hutchinson[7] relates a case of lupus which began at the age of 50, and was secondary to suppurating strumous disease of the sub-maxillary glands. He comments upon the unusual combination of lupus with any other tubercular manifestation.

Hallopeau[8] considers that the tuberculous character of a skin lesion can be decisively recognised by the following points: The possibility of transmitting tuberculosis by inoculation; the appearance of tubercle bacilli; and the production of alteration and of different eruptions by the action of tuberculin. He includes erythematous lupus among the tuberculous affections. His reasons for so doing are the following: (1) The occasional occurrence of adenopathy in the surrounding parts, the tuberculous nature of which can be demonstrated; (2) the reaction produced by injections of tuberculin.

Barclay[9] reports four cases of lupus treated with thyroid extract; in two cases a complete cure was obtained, and in the other two considerable improvement was noticed. He does not think that one can expect a complete cure, even if full doses be given, in a shorter time than one year. He found no serious effects from the use of the drug in youthful patients, but in those who had passed fifty years some irregularity in the heart's action was noticed; this, however, was easily controlled by reducing the dose and by giving some stimulant.

Nevins Hyde[10] considers that the differences between the several clinical forms of cutaneous tuberculosis may be explained by the quantity of micro-organisms present in any given case, by differences in the tissues on which the germs are implanted, and by the amount of exposure of the infected region. He stated that there is every reason to believe that, although the bacillus tuberculosis has not always been demonstrated in certain skin diseases, it will be found in the future. Amongst this group of probable tuberculous diseases, he includes: (1) Lupus erythematosis, (2) Bazin's disease, (3) lichen scrofulosorum, (4) the suppurative tuberculosis of Hallopeau, (5) a group of acneiform, sycosiform, and follicular disorders, (6) keloid, and (7) ulcus molle.

[1] Med. Bull., June, 1896, p. 210. [2] Am. Med. Surg. Bull., Sept. 19, 1896. [3] Am. Journ. of Med. Sci., July, 1896, p. 102. [4] Med. Week, June 25, 1896, p. 277. [5] Ed. Med. Journ., Nov., 1896, p. 472. [6] Ep. B.M.J., Aug. 29, 1896. [7] Clin. Journ., Aug. 5, 1896, p. 230. [8] Ditto, Aug. 12, 1896, p. 249. [9] B.M.J., Oct. 24, 1896, p. 1,200. [10] Lancet, Aug. 15, 1896, p. 466.

